# Ocular Biometry Features and Their Relationship with Anterior and Posterior Segment Lengths among a Myopia Population in Northern China

**DOI:** 10.3390/jcm13041001

**Published:** 2024-02-09

**Authors:** Linbo Bian, Wenlong Li, Rui Qin, Zhengze Sun, Lu Zhao, Yifan Zhou, Dehai Liu, Yiyun Liu, Tong Sun, Hong Qi

**Affiliations:** 1 Department of Ophthalmology, Peking University Third Hospital, Beijing 100191, China; linbobian@163.com (L.B.);; 2 Beijing Key Laboratory of Restoration of Damaged Ocular Nerve, Beijing 100191, China

**Keywords:** anterior segment length, posterior segment length, IOL Master 700, myopic patients, axial length

## Abstract

**Objectives:** The study aims to explore the ocular biometry of a myopic population in Northern China, focusing specifically on anterior and posterior segment lengths. **Methods:** This is a cross-sectional study. The medical records of 3458 myopic patients who underwent refractive surgery were evaluated. Axial length (AL), anterior chamber depth (ACD), lens thickness (LT) and other biometric parameters were measured using the IOL Master 700. The study determined the anterior segment length (ASL = ACD + LT), the posterior segment length (PSL = AL − ASL) and the ratio of ASL to PSL (ASL/PSL). **Results:** This study included 3458 eyes from 3458 myopic patients (1171 men and 2287 women). The mean age was 27.38 ± 6.88, ranging from 16 to 48 years old. The mean ASL was 7.35 ± 0.27 mm, and the mean PSL was 18.39 ± 1.18 mm. The ASL and PSL trends demonstrate an age-related increase for both genders, with notable gender-specific variations. Across most age groups, males typically exhibited higher ASLs and PSLs than females, with the exception of the 35–40 and 40–45 age groups. The ASL and PSL consistently increased with a rising AL. The AL strongly correlates with the PSL and negatively correlates with the ASL/PSL ratio. The ACD and LT moderately correlate with the ASL, but an increased LT does not imply a longer posterior segment. The CCT and SE show little correlation with axial eye parameters. **Conclusions:** Among Chinese myopic patients, a longer ASL and PSL were correlated with older age and the male gender. The AL strongly correlates positively with the PSL and negatively correlates with the ASL/PSL ratio. An elongation of the posterior segment may primarily account for an eyeball’s lengthening.

## 1. Introduction

The increasing prevalence of myopia, especially in Asia, is a significant public health challenge. Recent research, such as a study conducted by Gao et al., has emphasized a noticeable rise in myopia rates among university students. At Tianjin Medical University, the occurrence of myopia increased from 93.5% in 2017 to 96.2% in 2020, suggesting a rising issue in educational environments [1]. Although the axial length (AL) is a crucial measurement when investigating eye growth, advances in ocular biometry have added supplementary measures that provide a more comprehensive viewpoint. Research into ocular biometry has shed light on the relationship between the AL and other biometry parameters in myopic eyes. An investigation conducted by Miao et al. concerning the biometry of the anterior segment in cases of high myopia unveiled that, in addition to cataractous eyes, high axial myopia was linked to corneal flattening, increased total corneal astigmatism and anterior segment enlargement [2]. The investigations’ findings emphasize the complex correlation between anterior segment measures, the AL and the prevalence of myopia. This information provides insights for the formulation of therapeutic and public health policies targeted at tackling this pervasive disorder.

Two novel ocular biometric parameters, the anterior segment length (ASL) and the posterior segment length (PSL), have been applied to certain research in recent years [3]. The ASL is defined as the sum of the anterior chamber depth (ACD) and lens thickness (LT). It provides valuable insights into the structural components of the eye’s anterior portion. Recent research has significantly advanced our understanding of the anterior segment, particularly in the context of myopia and cataract populations. In the realm of cataract research, studies like those by Pakuliene et al. and Lei et al. have explored the relationship between the ACD and ocular biometry parameters in cataract patients, revealing insights into anterior chamber angle anatomy and its correlation with lens position and the AL [4,5]. These findings are crucial for predicting narrow angles and understanding the biometric changes accompanying cataracts. Several studies have highlighted the associations between anterior segment biometry and high axial myopia [6,7,8]. Nevertheless, the overall anterior segment dimensions of the eyes have not been the focus of most studies.

The PSL represents the length of the vitreous chamber. It is derived by subtracting the ASL from the AL [9]. The PSL is instrumental in understanding the elongation of the eye. In myopia research, the PSL is especially relevant as it provides quantitative insights into the elongation patterns of the eye, a key factor in the progression of myopia. Paritala et al. conducted a study focusing on the vitreous chamber depth (VCD), which may also be referred to as the PSL, and its relationship with the AL (VCD/AL) and discovered a highly significant link between the AL and the VCD in individuals with high myopia [9]. This study underscores the importance of considering both the AL and PSL while comprehending the progression of myopia. In the field of cataracts, research by Qi et al. revealed that a longer ASL and a shorter PSL were associated with older age and the male gender, and the PSL correlated positively with the AL across the study population, indicating the importance of the PSL in the biometric assessment of cataractous eyes [3]. In the work of Jian et al., it was found that eyes that are more keratoconic may have a shorter AL and PSL but a deeper ACD [10]. Moreover, for the PSL, there was a clear distinction between the various retinopathy of prematurity (ROP) stages, whereas for the ACD, there was just a little difference [11]. Eyes afflicted by central retinal vein occlusion (CRVO) may have a reduced PSL compared to unaffected eyes. This anatomical characteristic might potentially lead to a higher likelihood of congestion in the central retinal vein and artery within the lamina cribrosa, therefore increasing the risk of developing CRVO [12]. 

The findings from these studies suggest that a comprehensive approach to measuring ocular biometry, including the ASL and PSL, could bring some new insights. Examining the diverse biometric patterns of the ASL and PSL within different populations can aid in our understanding of how the anterior and posterior segments grow, identify high-risk individuals and provide individualized care. However, there has been no comprehensive investigation conducted on the distribution of the ASL and PSL in the Chinese myopia population. Building upon the foundation laid by prior research, our study aims to comprehensively explore the ASL and PSL in the myopic population of Northern China. In this study, we measured the ocular biometry, including the AL, the central corneal thickness (CCT), the ACD, the LT, the keratometry (K), the total keratometry (TK), the keratometry spherical equivalent (SE), the corneal astigmatism based on keratometry (DeltaK), the total keratometry spherical equivalent (TSE), the corneal astigmatism based on total keratometry (DeltaTK) and the white-to-white distance (WTW), of myopic people who visited a refractive surgery center in Northern China using IOL Master 700 (Carl Zeiss Meditec AG, Jena, Germany). As we delved into the ASL and PSL measurements using advanced biometric tools, we anticipated uncovering novel correlations and contributing to the growing body of knowledge that shapes myopia management.

## 2. Methods

This cross-sectional comparative study was conducted at Peking University Third Hospital, Beijing, China. The study was approved by the Ethics Committee of the Peking University Third Hospital (M2023687) and was performed in accordance with the tenets of the Declaration of Helsinki. Informed written consent was obtained from all participants after a detailed explanation of the study. 

### 2.1. Subjects

This study retrospectively reviewed medical records of refractive surgery, including corneal refractive surgery and implantable collamer lens (ICL) implantation, at the Peking University Third Hospital from November 2019 to September 2022. Patients with corneal opacity, lens dislocations, prior ocular trauma or operations or other ocular diseases that might influence the measurements were excluded. For each patient in this study, only one eye was counted. One eye was selected randomly for patients with both eyes eligible. In total, 3458 eyes of 3458 patients were included in this analysis.

The IOL Master 700 was used by skilled technicians to collect ocular biometric parameters. The technician visually verified the accurate fixation of the examinees on the fovea scan during each measurement. The standard deviation (SD) was automatically computed for each measurement of the AL, ACD and LT. If the SD for the AL exceeded 0.027 mm or for the ACD exceeded 0.021 mm, the device issued a warning indicating poor-quality findings. These data were discarded, and the measurements were repeated until consistent values were achieved.

The measurement of the AL was conducted by employing signals originating from the tear film and extending to the retinal pigment epithelium (RPE) of the fovea. The ACD was defined as the measurement of the distance between the anterior surface of the cornea and the anterior surface of the lens. The LT was defined as the measurement of the distance between the anterior and posterior surfaces of the lens. The ASL was determined by measuring the distance between the anterior surface of the cornea and the back surface of the lens. This value is obtained by adding the values of the LT and ACD. The PSL was determined as the difference between the AL and the ASL, representing the distance between the posterior lens surface and the RPE of the fovea. The ASL-to-PSL ratio (ASL/PSL) was also computed. In this study, we categorized all the eyes into six groups based on the AL measurements (19–22, 22–24, 24–26, 26–28, 28–30 and 30–32 mm) [5].

### 2.2. Statistical Analysis

The continuous data are shown as mean ± SD, while the categorical data are presented as the frequency for each category. The normality of the data for all ocular biometric parameters was assessed using the Kolmogorov–Smirnov (K-S) test. A skewed distribution was determined if *p* < 0.05. The Mann–Whitney U-test was used to analyze differences between two groups for continuous data, whereas the Kruskal–Wallis test was used to assess differences among more than two groups. The Pearson chi-squared test was used to compare differences in categorical data. The study used multiple linear regressions to analyze the relationship between the ASL and PSL, taking into account the AL, CCT, ACD, LT and SE as independent factors. Pearson’s correlation was used to evaluate the associations between the lengths of the anterior and posterior segments and ocular biometric data. *p*-values below 0.05 were deemed to be statistically significant. The analyses and visualizations were conducted using IBM SPSS v27.0 (Chicago, IL, USA).

## 3. Results

### 3.1. Characteristics

Table 1 shows the general and ocular biometric characteristics of this study population. This study included 3458 eyes of 3458 myopic patients (1171 men and 2287 women). The mean age was 27.38 ± 6.88, ranging from 16 to 48 years old.

**Figure 1:**
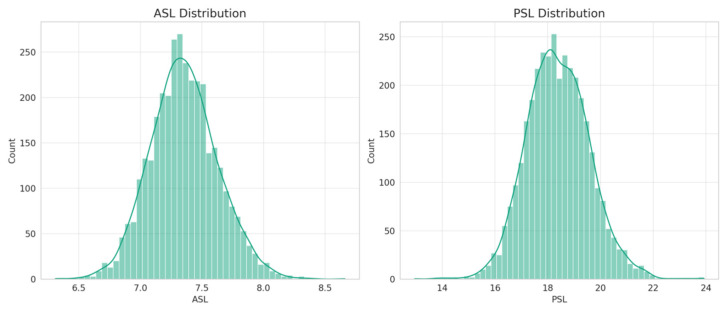
Distribution histograms for ASL and PSL.

The mean ASL in this study population was 7.35 ± 0.27 mm, the mean PSL was 18.39 ± 1.18 mm and the mean ASL/PSL ratio was 40.0 ± 3.0%. The distributions of the ASL and the PSL among this study population are shown in Figure 1. The histograms suggested bell-shaped distributions for both the ASL and PSL, albeit with minor deviations from perfect normality (Kolmogorov–Smirnov test, both *p* < 0.001). 

### 3.2. Comparisons of ASL and PSL Stratified by Age and Sex

**Figure 2:**
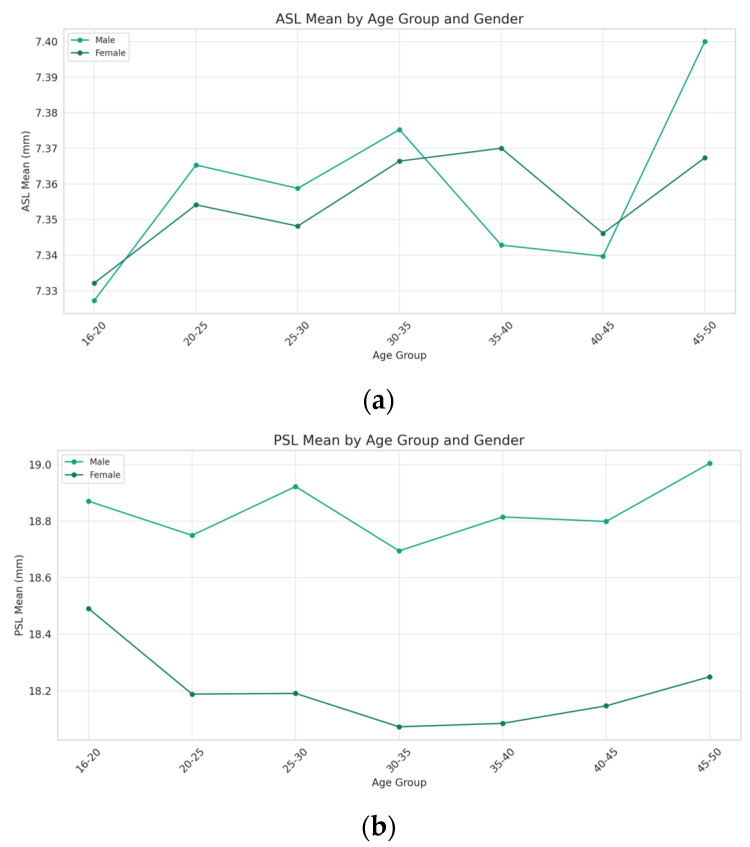
Comparisons of ASL and PSL stratified by age and sex. The (**a**) ASL and (**b**) PSL trends demonstrate an age-related increase for both genders, with notable gender-specific variations. Males generally exhibit higher ASLs and PSLs than females across age groups, except for the 35–40 and 40–45 groups. Males show consistent PSL growth, while females have a decrease until the 35–40 group, followed by a significant rise at 45–50.

There is a general increase in the ASL with age for both genders. However, this increase is not linear across the age groups. Notably, the ASL for the females shows a significant increase in the 45–50 age group after a period of relative stability in the preceding age groups. On the other hand, the males exhibit a peak in the ASL in the 30–35 age group, followed by a decrease and then another increase in the 45–50 age group. Overall, the males tend to have a higher ASL compared to the females, except in the 35–40 and 40–45 age groups (Figure 2a).

While the PSL also increases with age for both genders, the males display a more consistent rise across age groups. In contrast, the females exhibit a decrease in the PSL from the 16–20 age group to the 35–40 age group, followed by an increase in the 45–50 age group. Throughout the age groups, the males have a higher PSL mean compared to the females, with the gap widening noticeably in the 45–50 age group. However, due to the limitations in sample size for certain age groups, the statistical significance of the differences between genders could not be determined (Figure 2b).

### 3.3. Changes in ASL and PSL with AL

**Figure 3:**
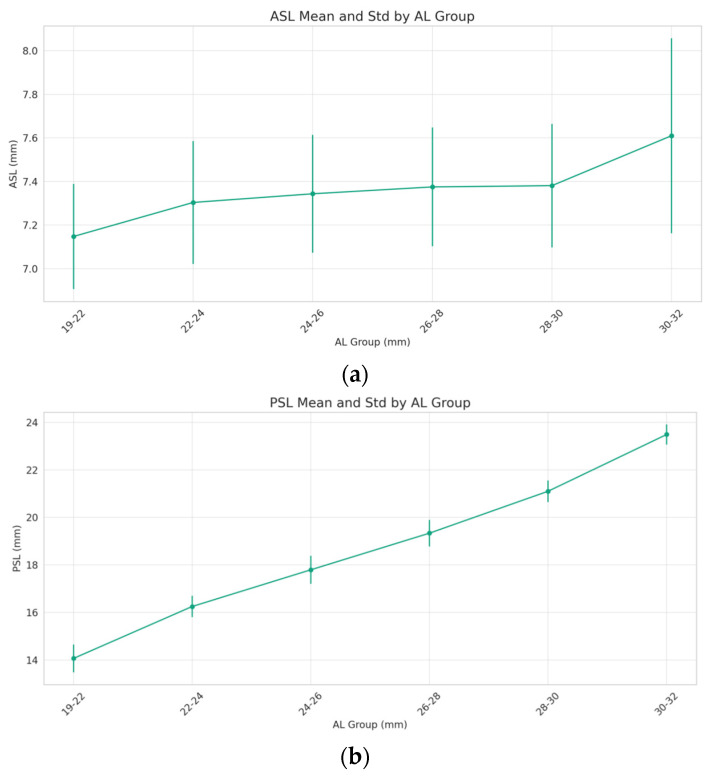
Changes in ASL and PSL with axial length (AL). The (**a**) anterior segment length (ASL) and (**b**) posterior segment length (PSL) consistently increased with rising AL.

The ASL presents a gradual increase as the AL extends from 19 mm to 32 mm. This relationship is not linear, as evidenced by a notable surge in the ASL for the AL 30–32 mm group. The variability within each AL group, represented by the error bars, suggests a consistent range of ASL measurements across most AL categories, with a pronounced increase in variability in the 30–32 mm group (Figure 3a). The PSL ascends steadily as the AL increases. This trend demonstrates a robust correlation, where a longer AL is associated with a greater PSL. The standard deviation within each AL category remains relatively uniform, implying a consistent relationship between the AL and PSL across the spectrum (Figure 3b). ANOVA test was conducted, indicating significant differences in the ASL and PSL measurements across the different AL groups (both *p* < 0.001). 

### 3.4. Correlation Analysis of Various Eye Parameters

**Figure 4:**
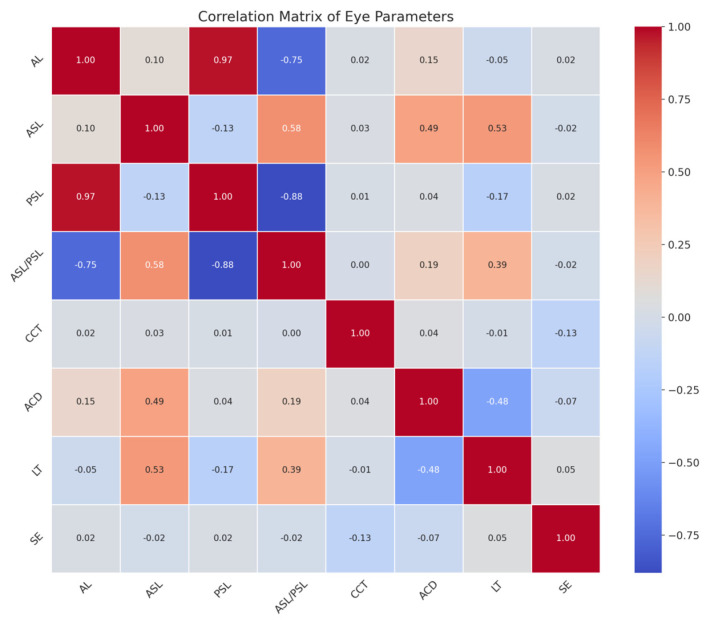
Correlations of ASL and PSL with other ocular biometric characteristics. AL strongly correlates with PSL and negatively correlates with the ASL/PSL ratio. ACD and LT moderately correlate with ASL, but an increased LT does not imply a longer posterior segment. CCT and SE show little correlation with axial eye dimensions. AL, axial length; ASL, anterior segment length; PSL, posterior segment length; ASL/PSL, the ratio of ASL to PSL; CCT, central corneal thickness; ACD, anterior chamber depth; LT, lens thickness; SE, keratometry spherical equivalent.

A correlation analysis was performed to identify the relationships between these parameters. A correlation matrix was generated using a heatmap to depict the strength and direction of the relationships. The heatmap provides a comprehensive overview of the correlation between various ocular measurements. Strong correlations are indicated by darker shades (Figure 4).

A strong positive correlation is observed between the AL and the PSL, as indicated by a coefficient close to one. Conversely, there is a strong negative correlation between the AL and the ASL/PSL ratio, denoted by a coefficient nearing −0.75. The ACD and LT both exhibit a moderate positive correlation with the ASL. The LT shows a strong negative correlation with the ASL/PSL ratio. As the LT increases, the anterior segment lengthens, but this does not necessarily translate to an increase in the posterior segment’s length. The CCT shows very little to no correlation with the other parameters, implying that the corneal thickness is independent of axial dimensions and segment lengths within the eye. The SE does not exhibit significant correlations with the axial dimensions.

## 4. Discussion

In this comprehensive study, we explored the ocular biometry of a myopic population in Northern China, with a specific emphasis on the ASL and PSL. The study included a diverse sample of individuals aged from 16 to 48 years with varying degrees of myopia. This demographic representation is reflective of the broader population in the region, providing a robust basis for our analysis.

The eyeball is anatomically separated into two segments: the anterior segment and the posterior segment. The anterior segment consists of the cornea, anterior chamber, iris and lens. On the other hand, the posterior segment is filled with the vitreous body, retina, choroid and sclera. The IOLMaster 700, which is based on swept-source OCT, offers an image-based measurement that shows the entire longitudinal section of the eye. We conducted a thorough examination where we precisely measured the ASL and PSL using the IOLMaster 700 [13,14]. 

Our findings revealed that the distribution of the AL and the calculated ASL and PSL values align with previous studies conducted among East Asian populations. The mean ASL was 7.58 ± 0.39 mm and the mean PSL was 17.12 ± 2.64 mm in a cataractous population in Shanghai [3]. This consistency suggests that the myopic population in Northern China shares biometric characteristics with other East Asian cohorts. On the other hand, the ASL in our research group was comparatively short. This might be attributed to the generally lower thickness of the lens in young individuals. However, the discrepancy in the ASL also indicates a lesser decline in the ACD compared to the increase in the LT as individuals age [15]. Furthermore, it is possible for there to be variances caused by systematic errors and environmental factors. The measurements of the ASL and PSL contribute additional layers of information, allowing for a more nuanced understanding of ocular biometry. Comparisons with the existing literature, especially in the context of myopia, will enhance our comprehension of these parameters’ significance in clinical practice.

The observed differences in the ASL and PSL as individuals age can be linked to physiological alterations in the anatomy of the eye throughout time. For both genders, the ASL and PSL trends show an increase with age. This could also be a reflection of how myopia develops in adulthood. Although myopia typically starts and progresses in childhood, it can also occur and develop during adulthood [16]. Myopia in adults needs to be continuously managed because a higher degree of myopia increases the absolute risk of myopia-related eye disorders and visual impairments. Sexually distinct characteristics can be explained by hormonal, genetic and environmental variables that affect the development and aging of the eyes [17]. The rise in the ASL with age, especially in females aged 45–50, may be attributed to hormonal effects on ocular structures, a notion that is substantiated by current evidence [18]. The gender disparities seen in the ASL and PSL distributions may suggest intrinsic anatomical and physiological variances, which are essential for tailored ophthalmic treatment [19]. These findings are consistent with research that indicates hormonal and biomechanical variables have a role in determining ocular dimensions.

Our analysis also revealed significant correlations between several eye parameters. The strong positive correlation between the AL and the PSL and the negative correlation between the AL and the ASL/PSL ratio highlight the interconnected nature of ocular biometry. The variations in the ASL and PSL across the different AL groups emphasize the need for individualized biometric assessments in refractive surgery and cataract management. This is particularly relevant for intraocular lens (IOL) power calculations, where accurate biometric data are essential for optimal visual outcomes [20,21]. The study’s findings could inform the development of more precise biometric models for IOL power calculations, especially in patients with extreme ALs [22]. The correlation between the PSL and the AL, especially in cases of high myopia, underscores the role of posterior segment elongation in the progression of myopia. This finding is consistent with the notion that myopia involves structural changes in the eye, not just refractive errors [23]. The study by Paritala et al. on the correlation of the VCD with ocular biometry in high axial myopia provides foundational support for this understanding [9].

The independence of corneal parameters such as the CCT and SE from axial dimensions is an important finding, suggesting that corneal properties may be assessed independently of AL variations. This has implications for the diagnosis and management of corneal disorders and refractive errors, particularly in myopic patients. The IOL-Master revealed corneal power measurements averaging 43.57 ± 1.45 D, with an associated corneal astigmatism of −1.31 ± 0.78 D. Furthermore, this study utilized the IOL Master 700, which was equipped with novel software, to assess both the traditional K and TK, which reflect the refractive power of both the anterior and posterior corneal surfaces. The measurements derived from the K and TK exhibit strong concordance. Comparable results were found in a study by Kommineni [24], emphasizing the reliability of IOLMaster measurements in assessing corneal parameters in myopic eyes.

In clinical situations, it is crucial to comprehend not just the AL but also the individual contributions of each part, which may be determined using ASL and PSL measurements. Advanced biometry instruments enable extensive measurements of the eye’s dimensions, therefore enhancing our comprehension of its anatomy. These characteristics provide doctors with a more comprehensive viewpoint for strategizing myopia prevention and refractive procedures, choosing intraocular lenses and handling different ocular disorders. Many effective strategies for preventing and controlling myopia have been developed recently; these include outdoor activities, low-dose atropine eye drops, multifocal spectacle and orthokeratology [25]. The prevention and management of myopia are major concerns for ophthalmologists as well as the general public. Future refractive observations of individuals at risk of myopia should focus more on the ASL and PSL in addition to the AL since the AL has a significant positive correlation with the PSL and a negative correlation with the ASL/PSL ratio. It is essential to identify these characteristics in order to create effective preventative measures and early treatments, which might help to slow down the evolution of myopia and decrease related consequences [26].The results of this study might have implications for tailored therapeutic treatments and the overall comprehension of myopia in Northern China. Refractive calculators currently in use for cataract surgery and phakic intraocular lens implantation typically employ regression or artificial intelligence to forecast the postoperative refractive state based on biometric parameters like the AL, K, ACD, LT, etc. [27]. The calculation might be more accurate if the parameters of the posterior segment of the eyes are taken into consideration. Furthermore, the findings of the study may stimulate additional inquiries into the genetic and environmental determinants that impact the ASL and PSL. Additionally, prior studies have described the characteristics of the PSL in patients with retinal diseases like ROP and CRAO [11,12]. Further investigation is essential to confirm whether the PSL has a distinct manifestation in common diseases like diabetic retinopathy or posterior vitreous detachment. The ASL, PSL and ASL/PSL ratio have potential for an in-depth explanation of the traits of pathological myopia and microphthalmia as well as the tracking of their occurrence processes.

Although the study provides helpful insights, it is crucial to recognize its limitations. This study primarily analyzes the biometric parameters measured by the IOLMaster 700. For a study on a myopic population, it is necessary to present the distribution of myopia diopters. However, due to the papery archiving of refractive results at this ophthalmology center, the large sample size made it difficult to collect and statistically analyze the refractive results. The use of a cross-sectional design hinders our capacity to identify causal linkages. Longitudinal research would provide a more comprehensive and detailed understanding of the development of myopia and its related alterations in the eye. These studies would focus on how these parameters evolve over time and their influence on ocular health and the advancement of diseases. Moreover, the study’s emphasis on a particular geographic area might limit the applicability of the results to different populations. Subsequent investigations should strive to incorporate a broader spectrum of individuals from various places in China to account for any disparities in ocular attributes. To gain a greater knowledge of the multifactorial nature of myopia, future research should consider including additional criteria like corneal biomechanics and retinal morphology. Further investigation is required to examine the fundamental processes that contribute to the variations in the ASL and PSL based on age and gender. Furthermore, investigating the genetic and environmental variables that contribute to differences in ocular biometry might improve our comprehension of myopia and other ocular diseases.

In conclusion, this research provides data on the ocular characteristics of the myopic population in Northern China. This study’s findings highlight the intricate relationship between ocular biometry parameters and their clinical significance. In this myopic population, male gender and older age were associated with prolonged ASLs and PSLs. The AL strongly correlates positively with the PSL and negatively correlates with the ASL/PSL ratio. The posterior segment’s elongation could be the main cause of the eyeball’s lengthening. There are no perceptible correlations between axial dimensions and the CCT or SE. The comprehensive ocular biometry provided may guide practitioners in optimizing treatment strategies for myopic individuals with diverse anatomical features. It is essential to identify these characteristics in order to create effective preventative measures and early treatments, which might help to slow down the progression of myopia and minimize related consequences.

## Figures and Tables

**Table 1:** General and ocular biometric characteristics in this study population.

	Total (*n* = 3458)
Age, years	27.38 ± 6.88
Sex, male/female	1171/2287
AL, mm	25.74 ± 1.18
ASL, mm	7.35 ± 0.27
PSL, mm	18.39 ± 1.18
ASL/PSL, %	0.40 ± 0.03
CCT, μm	539.23 ± 32.20
ACD, mm	3.67 ± 0.27
LT, mm	3.69 ± 0.27
SE, diopter	43.57 ± 1.45
DeltaK, diopter	−1.31 ± 0.78
TSE, diopter	43.56 ± 1.42
DeltaTK, diopter	−1.18 ± 0.72
WTW, mm	11.95 ± 0.42

AL, axial length; ASL, anterior segment length; PSL, posterior segment length; ASL/PSL, the ratio of ASL to PSL; CCT, central corneal thickness; ACD, anterior chamber depth; LT, lens thickness; SE, keratometry spherical equivalent; DeltaK, corneal astigmatism based on keratometry; TSE, total keratometry spherical equivalent; DeltaTK, corneal astigmatism based on total keratometry; WTW, white-to-white distance.

## Data Availability

The data presented in this study are available on request from the corresponding author.
